# A qualitative study of the acceptability of cognitive bias modification for paranoia (CBM-pa) in patients with psychosis

**DOI:** 10.1186/s12888-019-2215-3

**Published:** 2019-07-23

**Authors:** C. J. Leung, A. Fosuaah, J. Frerichs, M. Heslin, T. Kabir, T. M. C. Lee, P. McGuire, C. Meek, E. Mouchlianitis, A. S. Nath, E. Peters, S. Shergill, D. Stahl, A. Trotta, J. Yiend

**Affiliations:** 1 Department of Psychosis Studies, King’s College London, Institute of Psychiatry, Psychology and Neuroscience, London, UK; 2 Laboratory of Neuropsychology, The University of Hong Kong, Hongkong, Hong Kong; 3 The McPin Foundation, London, UK; 4 Health Service and Population Research Department, King’s College London, Institute of Psychiatry, Psychology and Neuroscience, London, UK; 5 The State Key Laboratory of Brain and Cognitive Sciences, The University of Hong Kong, Hongkong, Hong Kong; 6 South London and Maudsley NHS Foundation Trust, Bethlem Royal Hospital, Monks Orchard Road, Beckenham, Kent, BR3 3BX UK; 7 Department of Psychology, King’s College London, Institute of Psychiatry, Psychology and Neuroscience, London, UK; 8 Department of Biostatistics, King’s College London, Institute of Psychiatry, Psychology and Neuroscience, London, UK

**Keywords:** Cognitive bias modification, Interpretation bias, Qualitative research, Psychosis, Paranoia

## Abstract

**Background:**

Cognitive Bias Modification (CBM) has been used successfully as a computer-based intervention in disorders such as anxiety. However, CBM to modify interpretations of ambiguous information relevant to paranoia has not yet been tested. We conducted a qualitative investigation of a novel intervention called CBM for paranoia (CBM-pa) to examine its acceptability in patients with psychosis.

**Methods:**

Eight participants with psychosis who completed CBM-pa were identified by purposive sampling and invited for a semi-structured interview to explore the facilitators and barriers to participation, optimum form of delivery, perceived usefulness of CBM-pa and their opinions on applying CBM-pa as a computerised intervention. The interviews were transcribed and analysed using thematic analysis by researchers working in collaboration with service users.

**Results:**

Themes emerged relating to participants’ perception about delivery, engagement, programme understanding, factors influencing experience, perceived impact and application of CBM-pa. CBM-pa was regarded as easy, straightforward and enjoyable. It was well-accepted among those we interviewed, who understood the procedure as a psychological intervention. Patients reported that it increased their capacity for adopting alternative interpretations of emotionally ambiguous scenarios. Although participants all agreed on the test-like nature of the current CBM-pa format, they considered that taking part in sessions had improved their overall wellbeing. Most of them valued the computer-based interface of CBM-pa but favoured the idea of combining CBM-pa with some form of human interaction.

**Conclusions:**

CBM-pa is an acceptable intervention that was well-received by our sample of patients with paranoia. The current findings reflect positively on the acceptability and experience of CBM-pa in the target population. Patient opinion supports further development and testing of CBM-pa as a possible adjunct treatment for paranoia.

**Trial registration:**

Current Controlled Trials ISRCTN: 90749868. Retrospectively registered on 12 May 2016.

**Electronic supplementary material:**

The online version of this article (10.1186/s12888-019-2215-3) contains supplementary material, which is available to authorized users.

## Background

Cognitive models of persecutory delusions highlight the role of cognitive biases in the formation and maintenance of psychosis [1]. A growing body of research has investigated the potential of modifying cognitive biases in order to alter implicit processing in patients with psychosis. Empirical studies have shed light on the aetiological significance of information-processing biases that are congruent with the pathology content [2]. For instance, individuals with paranoid symptoms are more likely to interpret socially ambiguous situations as threatening than those without paranoia [3]. Such interpretation bias results in overexposure to negative information, which leads to the confirmation of persecutory beliefs, and in turn reinforces the pathological state [3, 4]. Altering these maladaptive biases may therefore break the vicious cycle of persecutory ideation and emotional distress exacerbating each other.

Cognitive Bias Modification (CBM) has emerged as a novel approach to systematically promote adaptive cognitive processing through computerised structured practice. CBM can be broadly categorized into CBM-A (attention training) and CBM-I (interpretation training), which respectively aim at attenuating attentional vigilance towards negative information (in the form of words/ faces) and modifying negative interpretation of emotionally ambiguous information. It does not rely on explicit instructions to induce change. Instead, to illustrate with CBM-I, it directly modifies negative interpretation biases by repeatedly exposing individuals to emotionally ambiguous information and implicitly constrains them to only resolve the information in a consistently positive manner [5]. Prior studies that simultaneously investigate the acceptability of CBM-A and CBM-I in anxiety disorders indicate that CBM-I is generally perceived as a logical and helpful procedure while CBM-A was reported to be repetitive and hard to remain engaged with [6, 7].

In contrast to interventions that require therapists, CBM can be self-administered, requires no specific skills or knowledge, and therefore could be widely and easily disseminated across various settings. Moreover, computerised delivery may be particularly beneficial in cases where paranoia hinders the development of trust in the therapist or reduces the therapeutic alliance [8]. CBM may also overcome the barrier of negative symptoms, which may deter patients from seeking face-to-face therapy [9]. Research evidence has demonstrated the efficacy of CBM-I in reducing pathological beliefs and symptoms within the affective disorder spectrum [10]. However, the application of CBM to people with psychosis is currently limited. Though CBM studies have been used to tackle anxiety symptoms in people with psychosis [11, 12] no study to date has applied CBM to modify interpretation bias specifically related to paranoia. There is also a paucity of studies that assess the credibility and acceptability of CBM as an intervention.

The current qualitative study complements the ‘Cognitive Bias Modification for paranoia’ (CBM-pa) feasibility, randomized controlled trial (RCT) [13]. It is of utmost importance to explore patients’ subjective experience of CBM-pa because adherence and engagement with treatment can fundamentally improve the outcome of intervention [14]. CBM-pa was developed based on previous empirical evidence of the presence of paranoia-relevant interpretation biases in people with clinical [3] and subclinical [4] paranoia as well as in those in the At Risk Mental State (ARMS) [15]. The purpose of CBM-pa was to encourage benign interpretations of emotionally ambiguous scenarios which could have both paranoid and non-paranoid interpretations. For example, a stranger staring at you could mean that they are admiring you in some way or that they have hostile intentions towards you; a group of people whispering as you walk past could mean that they have bad intentions towards you, or that they are simply discussing something private. To maximize the relevance to clinical paranoia, the study’s Lived Experience Advisory Panel (LEAP) was involved in creating and revising the content of the scenarios used in the study. The LEAP included a researcher (TK), as well as service users and carers with experience of psychosis and paranoid symptoms. The final ambiguous scenarios were developed from transcripts of service users’ real-life experiences of paranoia, plus some previously validated test items [3, 4, 15].

A number of CBM trials have evaluated acceptability through quantitative measures, such as attrition rates and questionnaires [7, 16]. However, quantitative data are insufficient to inform researchers about elements of the intervention that could benefit from further refinement to improve adherence and engagement. To the best of our knowledge, only two qualitative studies to date have examined patients’ subjective experience of CBM, focussing on social anxiety [6, 17]. Those findings derived important suggestions concerning how CBM should be promoted and delivered to the target population.

The present study aimed to explore qualitatively patients’ experience of receiving CBM-pa. Specifically, the acceptability of CBM-pa, facilitators and barriers to participation, optimum form of delivery, perceived helpfulness and impact of the intervention were examined using semi-structured interviews.

## Methods

### Participant selection and recruitment procedure

The complete sample of participants was recruited from a range of local sources, including: Institute of Psychiatry, Psychology and Neuroscience (IoPPN) and South London and Maudsley (SLaM) research registers; SLaM NHS Foundation Trust services; service users’ networks and voluntary sector organisations. Eight participants who completed CBM-pa were recruited to the current qualitative study. Participants were aged 18 to 65 and diagnosed with clinically significant paranoid symptoms by a trained clinical psychologist (AT) using the *Structured Clinical Interview for DSM-5* (SCID-5) [18]. To assess the severity of psychotic symptoms, the Positive and Negative Syndrome Scale (PANSS) [19] was used. Only those scoring 3 or above on Item 6 (suspiciousness/ persecution) were included in the study. Participants also had to display a baseline interpretation bias on the eight-item version of the Similarity Rating Task (SRT) by scoring 1 or less. The SRT is a standard measure of interpretation bias which depicts ambiguous social scenarios that permit either paranoid or non-paranoid interpretations [4]. Scores on the SRT can range from − 3 to 3, with a lower score indicating greater negative interpretation bias [3]. This cut-off was adopted to ensure those taking part in the trial had appropriate levels of pre-existing negative interpretation bias (the target mechanism of the intervention). Additionally, participants, for whom it was relevant, had to be stable on medication for at least 3 months and expected to be so throughout the study duration. This was an ethical and practical requirement to ensure participants remained in a sufficiently stable mental state to participate in the study without undue risk and to ensure symptoms were sufficiently controlled to permit full engagement with CBM-pa [20]. Participants were required to have the capacity to consent (assessed by CM and AT) and to speak English fluently to be eligible for the interview. Exclusion criteria were severe cognitive impairment, illiteracy, major physical illness (such as cancer, heart disease, stroke) and major substance or alcohol misuse (screened by using SCID-5). Those who were receiving, had recent experience (in the last 3 months) or expected to receive psychological therapy targeting paranoid beliefs were also excluded. Details of the trial methodology are published in a separate paper [13].

Qualitative interviews were conducted after the end of all quantitative data collection (i.e. after the final three-month follow up assessment). The eight participants (24%) were anonymously shortlisted from the 30 participants of the treatment arm by two members of the research team unfamiliar with the participants (JY and TK). The participants were purposively selected to represent the sample variation in the following priority order: gender, severity of paranoia, severity of bias, age and ethnicity [21]. In doing so we hoped to explore the applicability of CBM-pa as an intervention to participants of different backgrounds and characteristics. Despite a relatively small sample size, participants did represent almost one quarter of those who received the intervention. We intended that this, together with purposive sampling, would ensure data saturation was achieved. At a participant level data saturation was ensured by ending the interview when no additional new information was forthcoming. The selected participants were de-anonymised, contacted and invited to interview. One participant chose not to take part and was replaced. Participant characteristics are shown in Table 1.

**Table 1:** Demographic characteristics of participants

Characteristics	M (SD)	Number of participants
Gender		
Male		5
Female		3
Age		
30–39		2
40–49		3
50–59		3
Ethnicity		
Black-British		2
White-British		2
White-Irish		1
Pakistani		1
Indian		1
White and Asian		1
Baseline PANSS item 6 score	4.13 (0.83)	
Baseline SRT Bias score	0.27 (0.80)	

*Abbreviations*: *PANSS* Positive and Negative Syndrome Scale, *SRT* Similarity Rating Task

### CBM-pa programme

**Fig. 1:**
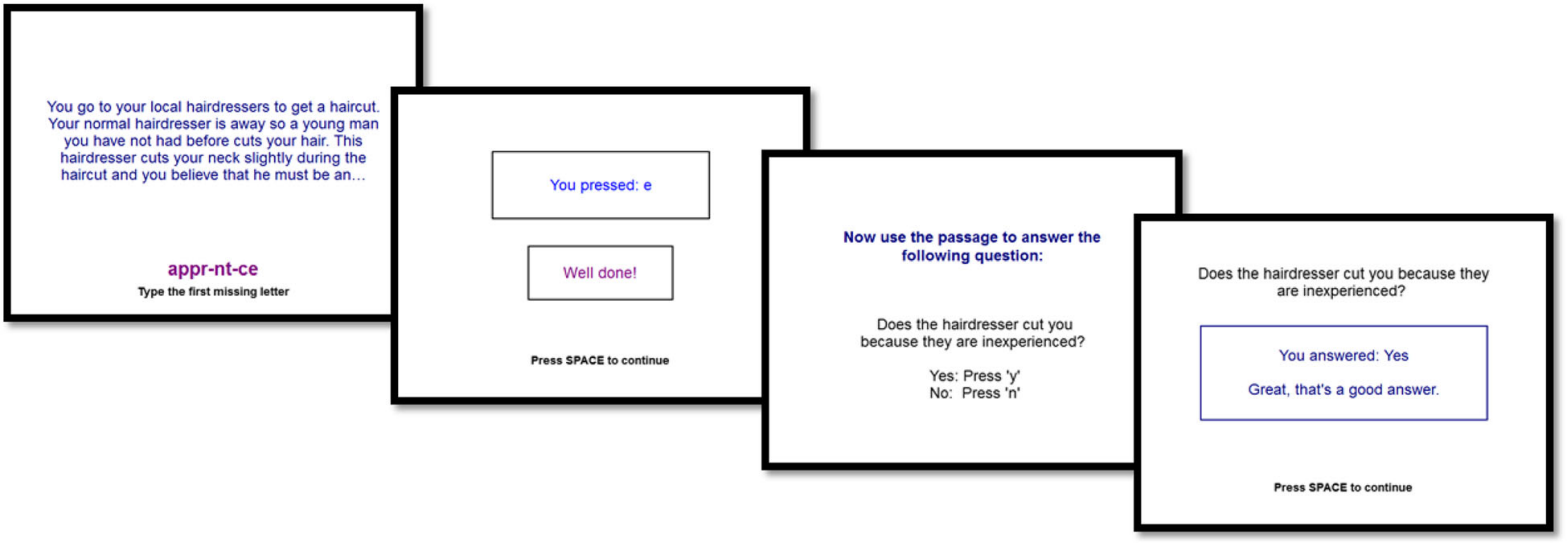
Example of CBM-pa intervention item

CBM-pa was delivered via computer and was self-administered at King’s College London. Patients were presented with text depicting ambiguous scenarios, which could be interpreted in a paranoid or benign way. See Fig. 1 for an intervention trial example. After patients read the scenarios, they were asked to complete a word fragment (frame 1 and 2 of Fig. 1) and comprehension question (frame 3 and 4), both of which implicitly encourage participants to interpret the ambiguous scenario in a manner that promotes helpful beliefs about themselves and others. The computerised programme contained 240 unique training items, delivered over six weekly 40-min sessions. Further details of the CBM-pa programme are described in the protocol paper [13].

### Interview procedure

A semi-structured topic guide, consisting of 10 main questions, developed in collaboration with the LEAP group, was employed to explore the acceptability of the CBM-pa intervention (see Additional file 1). It covered participants’ experience and perception of structure and format, as well as perceived helpfulness and impact of the intervention. Leading questions were avoided to minimize the impact of researcher bias. Following written informed consent, participants were shown a 10-min demonstration of the CBM-pa computer task as a reminder of the intervention. Face-to-face interviews were conducted at King’s College London with one lead researcher asking questions based on the semi-structured topic guide. With participants’ prior approval, a second interviewer was present to add additional prompts to further explore participants’ responses. Upon completion, participants received £30 worth of vouchers or cash for taking part in the interview. All interviews were audio recorded and transcribed verbatim into an Excel file.

### Data analysis

A qualitative descriptive study design was used to reflect the experience and opinions of participants that is closest to the data collected. Qualitative description adopts low-inference interpretation and thus the result ensures greater fidelity to participants’ accounts [22].

By taking the stance of post-positivist, we acknowledged that while we assume to present an objective reality, our role as researchers in the study may have affected the questioning, interpretation and co-creation of meaning [23].

We had a number of pre-conceived aspects of experience we wished to ask participants about and we therefore used elements of framework analysis [24] to address these questions and map the relevant data. However, we did not wish to confine ourselves to these questions, nor fit the data only into an a priori framework. We therefore combined this with a thematic analysis [25] of the data to identify emergent patterns or themes from the entire qualitative dataset. Most of the codes and all of the themes were not pre-determined by the a priori questions. The iterative analytical process drew on Braun and Clarke’s [25] six-step framework – familiarize with data; generate initial codes; search for themes; review themes; define themes and write up. Most researchers involved in data analysis and coding (JY, CM, AF, AN, CL) had training at master’s level or above in qualitative research methods.

Prior to analysis, AF and AN transcribed the interviews. At the familiarization stage, all researchers independently reviewed transcripts to develop an overview of the main ideas. Key aspects related to the research questions were identified and generated as initial codes. A coding framework was formed with the consensus of AF, AN, JY and JF. The data from each interview were then assigned, within Excel, to relevant codes from the agreed framework by AF and AN. New codes were generated if the data did not fit into the initial codes. To increase rigour in the process of analysis, the researchers referred back to the transcripts to ensure that the codes identified were applicable to the original data. In addition to AF, AN, JY and JF, the LEAP members and CL were involved in reviewing codes and integrating them together to form themes. Triangulation of themes was undertaken through extensive group discussion.

In terms of reflexive accounting, most researchers, based on previous research evidence of CBM-I [26] had the prior belief that CBM-pa would be beneficial in reducing participants’ interpretation bias. To minimise the effect of potential bias, researchers were specifically cautious in looking for refutational data during the analysis. Researchers also kept reflexive notes during the discussion and were aware of the similarities and differences among themselves and LEAP members in approaching the findings. Indeed, LEAP members offered perspectives and insights that the research team might not have identified.  For example, in the choice of theme labels, we were reminded to avoid terms perceived as undesirable, stigmatising, jargonistic or unnecessarily complex. Thus, theme structure and content were defined and refined until a final consensus was reached. Reporting of this study adheres to the CONSORT guidelines (where applicable [27]) and COREQ guidelines [28].

## Results

**Fig. 2:**
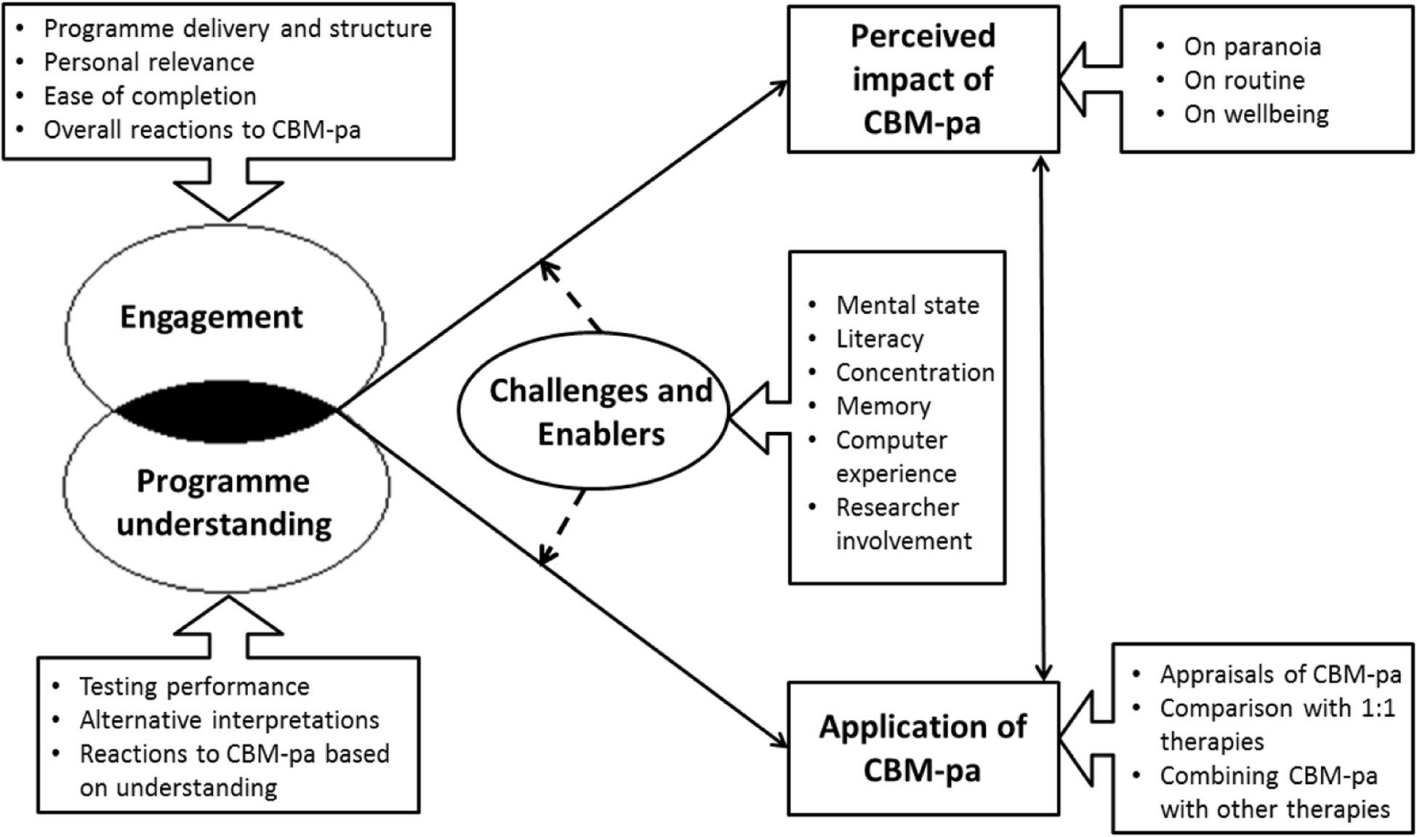
The conceptual relationship of themes. Figure 2 is a conceptual diagram which illustrates the relationship of the themes. Engagement and Programme understanding are inter-related and influence each other. These two factors could each affect the perceived impact of CBM-pa and the application of CBM-pa as a clinical intervention. But those who are both engaged and understood the purpose of the programme (shaded area) are more likely to find CBM-pa beneficial as an intervention. The potential clinical application of CBM-pa depends on its perceived usefulness. But it is also directly affected by participants’ engagement and level of understanding. In turn, participants’ suggestions on the application of CBM-pa could potentially augment the perceived impact of the programme. Challenges and Enablers which included participants’ individual characteristics could moderate the influence of Engagement and Programme understanding on the perceived impact of CBM-pa and its clinical application. For instance, even if participants found the design of CBM-pa engaging, their intrinsic mental state and ability to concentrate could affect the overall effectiveness of CBM-pa

Thematic analysis yielded five overarching themes – Engagement; Programme understanding; Challenges and Enablers; Perceived impact of CBM-pa; Application of CBM-pa and their corresponding sub-themes. Figure 2 depicts the relationship of the themes and the sub-themes are listed under each theme accordingly.

### Theme 1: Engagement

This theme depicted how participants found the intervention and how much they engaged in it. It covered factors that may affect participants’ engagement towards CBM-pa. It included individuals’ perception towards the delivery and structure of the intervention, the relevance of the scenarios to their own day-to-day lives as well as how easy or difficult they found the intervention was to complete. This theme also encompassed their overall reactions to CBM-pa. In general, participants were positive about CBM-pa and were engaged with the intervention.

### Programme delivery and structure

All participants indicated that the illustration and length of the text in the computerised task was clear and adequate.

*“Big bold and clear. Yeah, there was no need for braille or big lettering.” (Participant 4).*

The participants found the task instructions to be clear and easy to understand. Only a few people required further explanation and were subsequently able to work on their own. *“It was clear and understandable, pretty basic.” (Participant 4).*

*“Urm, I wasn’t so sure then, but once I got explained the procedure, then it wasn’t a problem. But anything new to me has to be spelt out to me so I can get the urm ...- get the hang of it but erm, once explained it wasn’t as I said a problem at all. I seemed to catch on quite quick” (Participant 3).*

Participants reported contentment with the structure, length and frequency of the intervention sessions.

*“I think it’s just right, normally when I am doing something that is tedious I feel a pain in my back coming on and I didn’t get that …*” *(Participant 8).*

*“One session a week yeah. Naa it was just the right amount. Yeah it was just right. Just right and I was looking forward to continue.” (Participant 3).*

### Personal relevance

Participants indicated that the scenarios presented corresponded to their real-life experiences. They could relate to the feelings as if they were in those situations. There was a pleasant feeling of being understood while doing the task, which was not obtained elsewhere in life.

*“I could relate to some of ‘em, ‘cus some of ‘em were everyday tasks that you do yourself at least once a week if not, half a dozen times a week” (Participant 4).*

*“I think I could relate to all of them, yeah I don’t feel as though I had a big issue relating to things … cause I am out there doing stuff and I have worked all my life, so I am aware of a lot of situations umm I could relate to all of those scenarios that I remember anyway,” (Participant 6).*

*“It felt really really good, I don’t know what this … it felt like someone had read through my files and actually realise that this guy’s having problems, we can offer him something you know, and that they realise that I was having problems when no one else had …*” *(Participant 8).*

### Perception towards ease of completion

CBM-pa was generally regarded as easy and straightforward to complete. Some participants said that more effort was needed to interpret the ambiguous scenarios from another perspective but found the overall experience to be acceptable.

*“Erm, the questions were interesting, it was quite easy to like you know put in like the letters and stuff like that, so I found it enjoyable.” (Participant 1).*

*“It was quite easy because I didn’t spend too long on the tasks and I think I answered most of them correctly after the first couple of sessions …*” *(Participant 8).*

*“My brain doesn’t think that way, so it was kind of- the effort involved in lifting something heavy to attempt to see it from that other point of view” (Participant 2).*

### Overall reactions to CBM-pa

Most participants described their experience of the intervention as enjoyable, interesting and helpful. While some participants reported having found the programme slightly laborious and repetitive, a few felt a bit disturbed for seemingly being forced to accept an alternative explanation to the ambiguous scenarios.

*“Made you think … I like to be challenged so I found it interesting … yeah” (Participant 6).*

*“Erm, it’s been really interesting to see my perception of reality versus what actually – actually putting some doubt into my perception of reality and seeing where there could be problems.” (Participant 2).*

*“It was okay, quite- quite straight forward … bit tedious, bit tedious and laborious.” (Participant 4).*

*“Felt like kind of a bit disturbing, it’s like they want me to maybe think the other way.” (Participant 5).*

### Theme 2: Programme understanding

This theme illustrated participants’ understanding of the purpose of CBM-pa. It highlighted that all individuals considered CBM-pa was a test of performance. But some of them understood the intervention component in the programme. This theme also encompassed participants’ subsequent reactions towards the programme based on their understanding.

### Testing performance

CBM-pa was unanimously perceived as a test, comprehension task or a concentration/ memory task.

*“Oh the English comprehension? It’s like an English comprehension in reading the phrase … -phrases or sentences and umm answering the questions. It’s like an English comprehension. Like when I used to do English comprehension.” (Participant 3).*

*“Yeah I did, it’s ummm it’s to do with my memory isn’t it? I think umm did I remember what I said the first one, and you have to remember, like was I waiting for the bus or wasn’t I waiting for the bus you know something like that.” (Participant 7).*

### Alternative interpretations

While all participants expressed the test-like nature of CBM-pa, some quotes within this theme showed CBM-pa was also considered as an intervention. A number of participants understood the task to be modifying paranoid thoughts, training emotional resilience, or assessing their confidence level.

*“Umm made you think of you being paranoid sometimes perhaps … and no you shouldn’t do it … you get a negative response from the computer just letting you know that’s not right … so it’s a good way to build up some positive imagery” (Participant 6).*

### Reactions to the programme based on understanding

Participants’ perception of their performance appeared to influence their experience of CBM-pa. Performing well made individuals feel good while perceived poor performance resulted in feelings of self-doubt.

*“It mattered whether you were right or wrong. You had to understand the passage, then when you got the question, was it yes or no? When you press the yes or no and you got it right, it was sorta like yeah! Like I understood, I’ve passed the test, I’ve done the task. Nobody likes to be wrong, do they?” (Participant 4).*

*“Erm it does- it makes me feel less, that I’m not doing good enough, stuff like that so.” (Participant 1).*

In response, some participants highlighted the need to emphasize CBM-pa is not a test, to minimize the negative impact on individuals when they answered “incorrectly”.

*“They need to be told at the beginning that it’s not a test, it’s a therapeutic intervention, and you can tell me what you feel it was assessing at the end if you like … so leave it for them to decide what it was all about” (Participant 6).*

Whereas, for those who interpreted CBM-pa as an intervention (as well as a test) they were positive about its impact.

*“Yeah intervention is suitable yeah … yeah … I liked doing it, it was good and as I got used to an idea I got to do difficult questions ( …*) *I tried to memorise things a bit more …*” *(Participant 6).*

### Theme 3: Challenges and enablers

This theme covered factors that could either present a challenge, or be facilitatory, to participants while taking part in CBM-pa. Factors were related to the individuals themselves, including their state of mind during the intervention, their memory capacity, literacy and concentration level as well as their computer experience. Researcher involvement during the intervention was also considered under this theme.

### Mental state

Some participants reported a feeling of nervousness arose from the need to adapt to a new environment and their concern about performance. Their level of alertness was also reflected as another factor that influenced the programme completion.

*“I think in the beginning the first couple of weeks it’s a bit nervous, you’re here to well, you don’t want the person or persons to know that you cannot spell properly, but I think my spelling is all right, just need a bit more concentration” (Participant 7).*

*“Well, I’d like to think that alertness comes into it. I didn’t feel as alert today maybe it’s because of intoxication I don’t know, I didn’t- or nervousness” (Participant 4).*

### Literacy

A few participants reported that reading the scenario passage was a challenge for them, but they were able to manage it by rereading the passages.

*“It took me a few seconds more to read it through ‘cus I had to read it through sometimes twice to get the theme.” (Participant 3).*

### Concentration

Most participants stated that they had difficulty concentrating during completing CBM-pa. They mostly ascribed their difficulties to the influence of pathology, medication or alcohol use.

*“Sometimes I can do it for a while then I lose concentration ‘cus I’m thinking stuff like ‘ahh are they watching me or something’, ( …*) *I’m just like being mindful of people around me and it stops me from answer [ing] the question or reading the sentences or passages...” (Participant 1).*

*“I’m always daydreaming and starring and, it’s just a habit I got with the medicine I’m taking.” (Participant 3).*

### Memory

Almost all participants reported worry about remembering the content of the scenarios. Some of them explained that their memory was being affected by poor concentration during the task.

*“Yeah … yeah I think I got them all right … it’s just the end bit where all the questions come about umm memory umm difficult to remember all the scenarios” (Participant 6).*

### Computer experience

A few participants noted that they were not familiar with computer usage which made completing the tasks difficult initially, but all of them were able to manage it over time.

*“I think the first couple of sessions was a bit difficult for me cause as I said, I do the computer but that task I have never done before, so it takes time to get used to it, but as you have seen today I have just done it.” (Participant 7).*

### Researcher involvement

Several participants mentioned feeling wary/ being watched when the researchers were present, though regarded them to be friendly and supportive. While participants acknowledged the researchers’ provision of timely support, they admitted to being more relaxed and focused once they were left alone with the task.

*“Sometimes when people are around me I can’t focus on what I’m doing, ( …*) *it just makes me a little paranoid.” (Participant 1).*

*“I felt more relaxed when you left the room but umm felt more confident when you was there in case I wanted to ask anything … cause I was on my own, I could just get on with it.” (Participant 7).*

*“Naa you just get deeply involved in the computer. It doesn’t matter who’s sitting there and to tell ya the truth, people who [do] the research sit in the background very quietly” (Participant 4).*

### Theme 4: Perceived impact of CBM-pa

This theme outlined the perceived impact of CBM-pa on participants’ paranoia, daily routine and wellbeing. It described individuals’ awareness/ recognition of paranoid thoughts, ability to challenge those thoughts and think of alternative explanations of situations. The theme also discussed the impact of the intervention on participants’ daily functioning and wellbeing, which included their mental state and relationship with others. All participants reported that CBM-pa had to a certain extent improved their overall wellbeing.

### On paranoia

Those who understood CBM-pa as a psychological intervention expressed improved insight towards their habitual manner of processing. They claimed to have learned ways of challenging their own thoughts and reacting positively instead of jumping to a negative or paranoid conclusion.

*“It helps you not to be so paranoid because you don’t know what other people are actually thinking. ( …*) *It made you recognise that the way you’re thinking might not be that - shouldn’t be too paranoid about … you know certain situations.” (Participant 1).*

*“( …*) *umm before I’d done this study I didn’t actually know how much … how much I am suffering with paranoia, because of the situations I was playing on the computer task, actually made me realise how I was reacting in real life to certain situations or maybe overreacting or not reacting in positive way” (Participant 8).*

Even for participants who commented that they had the feeling of being forced to change their preferred manner of interpretation, they nevertheless found CBM-pa to be helpful in uncovering their tendency towards biased processing.

*“Felt like kind of a bit disturbing, it’s like they want me to maybe think the other way” (Participant 5).*

*“I think when I was doing these tasks … I think I tend to agree with the alternative with my thoughts actually … I was challenging my thoughts ( …*) *I think it was starting helping me” (Participant 5).*

There were participants who admitted to being shocked at the frequency of harbouring paranoid thoughts and interpretations. Although the newfound awareness gave rise to feelings of self-doubt, it instilled hope to the participant in addressing the symptoms.

*“I have to make a lot of decisions and to realise that I might not have the insight that I thought I had is absolutely terrifying. Like how many people have I- have I made the wrong decision and hurt other people” (Participant 2).*

*“It was quite hopeful as well in that if I’m noticing all these things aren’t quite right at least that shows where I’ve got the problems. Erm and that there is hope to change it.” (Participant 2).*

### On routine

Participants noted that routinely attending CBM-pa sessions was another benefit of the intervention. Even those who did not perceive the programme as an intervention expressed their enjoyment in coming for the weekly session and meeting researchers as part of their established routines.

*“Think I was quite stable … yeah because I knew what I was coming to do every single week, yeah that was a good thing about it, I really like that … it was in a way to focus and reflect upon what I have been doing” (Participant 8).*

### On wellbeing

Some participants reported noticing an improvement in their wellbeing after completing CBM-pa. Besides gaining more self-confidence, they felt that they had better relationships with their family and friends as they learnt to show more understanding of others’ situations.

*“As I said I have gained knowledge, I can do this again, cause I do research when I am asked for it umm but not always it depends when I get it, umm it’s … I have gained confidence, I know that if I ever get shown that for research I know what I am going to do …*” *(Participant 7).*

*“Well I have a more lenient approach especially towards (girlfriend), and apart from my dad I have more lenient approach and self well-being and understanding situations” (Participant 3).*

### Theme 5: Applications of CBM-pa

This theme illustrated participants’ perception concerning the development of CBM-pa as a formal therapeutic intervention for paranoia. Advantages and disadvantages of CBM-pa and other existing one-to-one therapies were identified. Opinions centred on the pros and cons of human interaction in therapy and treatment. Most participants supported the combination of CBM-pa and elements of talking therapy, or some form of human interaction.

### Appraisals of CBM-pa

The reported advantages of CBM-pa focused mainly on the characteristics of the computerised task. Participants noted that working with a computer can avert dealing with human emotions, which they regarded to be difficult to handle. They also reported an increased sense of autonomy compared to receiving advice from mental health professionals. In addition, they regarded CBM-pa to be quick, more specific and focused than talking therapies.

*“Yeah because with humans you deal with emotions which I find it difficult … with computer you’re not” (Participant 5).*

*“Yes, here it’s a more formal assessment here, so you can have your say and initiate. But with the CPN and the doctors, if you have a problem, and you try to explain to them your problem and you feel touched by it, they classify it as part of your illness” (Participant 3).*

*“On the computer, you’re more focused on your task. So they asked you a question, yes or no answer, asked you to put yourself in that situation [and] give the right answer, whereas when I am talking, you get around the houses till you get to the right answer” (Participant 8).*

As for disadvantages, participants sometimes felt the need for a human connection, for example, to access empathy and support for any unresolved issues raised by engaging with the scenarios.

*“There’s- if you cry there’s a human being. I … I don’t know why that has a human connection, but it does because there’s someone there who can say yes, I understand why this is hard. But the computer does not see why that’s hard.” (Participant 2).*

### Comparison with one-to-one therapy and treatment

Participants compared CBM-pa to psychological therapies such as CBT. Their appraisals of one-to-one therapy demonstrated conflicting ideas about the pros and cons of human interaction in therapy. On one hand, they valued the human connection in talking therapy. Treatment direction and pace can also be tailored according to the participants’ progress.

*“Well I suppose [when] I was having one-to-one, it was tailor fitted around me whereas this is a lot of questions and everyone gets the same question. So umm it didn’t respond to immediate needs in the same way as a CBT practitioner would if you were face to face with them” (Participant 6).*

On the other hand, participants admitted their concern about interacting with mental health professionals. They expressed the difficulty to verbally express their thoughts and were worried that their responses may be incorrectly labelled as symptoms.

*“Also, there’s a lot of fears with doctors because you mention one thing and its ‘ohhh I’ve got this special drug here. So there’s this whole layer of emotions and this whole layer of complexity, whereas with a computer there isn’t. It’s me, it’s the computer” (Participant 2).*

### Combining CBM-pa with other therapies

Most participants favoured the idea of combining CBM-pa with elements of human interaction or talking therapies. They considered the involvement of another person would provide encouragement and feedback to enhance their understanding of the intervention and maximize its impact on daily life.

*“Feedback on the way you’re supposed to answer it, give encouragement in that way. And if they get it wrong, explain to them why it’s wrong, that would help. And then you know, think more in a way that is not causing them to think [in] that way- in the wrong way again” (Participant 1).*

Finally, a number of specific practical suggestions for changes to the CBM-pa intervention arose during the qualitative interviews and these are listed in Table 2 (a detailed version with participants’ quotes is available in Additional file 2).

**Table 2:** Practical suggestions for the future development of CBM-pa based on participants’ comments

Category	Suggestions
Appeal of CBM-pa	• Add audio and visual (video/ picture) display that matches with the scenario to enhance participants’ attention and sense of personal relevance while working on the task
Programme structure	• Implement CBM-pa for a longer period (twice a week and up to 6 months) to consolidate learning and sustain its impact • Incorporate alternative activities in between the CBM-pa task to sustain interest • Allow extra time for response • Give participants the option of completing CBM-pa at home
Content of scenarios	• Tailor the content of scenarios to increase personal relevance to participants. For instance, add a job interview scenario for those who would like to find work.
Being informed	• Give an introduction of the intervention in advance and provide explanation of the rationale
Human contact	• Offer means of contact in case emotional support is needed

## Discussion

### Main findings

To our knowledge, this is the first study to report the perspectives of patients with psychosis towards their first-hand experience of receiving CBM. The visual display and structure of the programme sessions received positive feedback. Participants reported engaging well with the ambiguous scenarios and achieving new insight into their own related patterns of thought, in some cases translating this into practice. This is contrary to previous literature which found CBM-I to be impersonal [6]. Using scenarios that were generated by service users and reflected their real-life experiences of paranoia is likely to have contributed to this positive finding. In general, most participants described CBM-pa as an easy, straightforward and enjoyable task. Occasional comments echoed the limitations of CBM reported by previous studies (e.g. repetitive, laborious [6, 7]), and a few participants reported feeling slightly disturbed and expressed difficulty accepting the benign explanation of the ambiguous scenarios. This was, however, expected among patients with paranoia given fixed false beliefs being the major symptom characteristic [29]. In fact, their struggle reflected that CBM-pa had at least prompted participants to try to adopt an alternative perspective, which was the main purpose of the intervention. Moreover, some of these participants recognised the benefit of CBM-pa in helping them gain insight into their tendency to make paranoid interpretations.

Most participants considered CBM-pa as a test of performance. Responding correctly in the task resulted in a feeling of competency and mastery, which is intrinsically motivating. Conversely, making ‘wrong’ task responses evoked negative reactions, such as self-doubt. Some participants reflected that they would appreciate being briefed about the content and purpose of CBM-pa before starting. This converged with Beard’s findings [6], in which participants felt the need to understand the purpose and relevance of the task to their anxiety. However, it is crucial to consider the pros and cons of explaining the rationale of CBM in advance, given the contrasting findings in the field. Mitchell and Gordon [30] found that participants’ ratings of computerised CBT improved after demonstration of the trial, but in Beard’s study [6], CBM was perceived to be less credible after participants experienced the trial. Kuckertz et al. [17] found that patients’ initial perception of CBM-A predicted both anxiety reduction and the extent to which attention bias was modified. These studies demonstrate the varying impact of prior expectation on perceived helpfulness and actual outcome. Furthermore, the suggested mechanism of action underlying CBM-pa is the implicit learning of a benign production rule driving interpretation [31]. Therefore, explicit understanding of the rationale behind CBM should not be necessary in order for the intervention to have beneficial effects. On the other hand, in our study, those who did understand the task as an intervention, found CBM-pa to be particularly helpful in improving their insight into paranoid ideations and awareness of alternative explanations for emotionally ambiguous situations. Overall, we suggest that future work should, as a minimum, provide recipients with an explicit rationale which contextualises CBM-pa as an intervention rather than a performance test.

A number of challenges and enablers that influenced participants’ experience of CBM-pa were identified. They included individual factors, such as participants’ mental state and memory capacity as well as the external factor of researcher involvement in the session. Some of the noted challenging factors appeared to be particularly difficult for participants with paranoia. For instance, some participants reported a feeling of being watched by the researcher and expressed concern about being judged for their task performance. Both the side effects of medication and cognitive impairments in attention and memory (evident in patients with psychosis [32, 33]), also affected the ability to concentrate and remember details of the scenarios. However, it is important to note that a certain degree of perceived difficulty in remembering the passages is not only expected but necessary, in order for the intervention to work as it does [23].

All reported that completing the programme had to a certain extent improved their overall wellbeing. In reviewing the application of CBM-pa, participants compared it with other therapies. Unlike traditional talking and pharmacological therapy in which doctors or therapists usually take the lead, CBM-pa is self-administered and self-paced. This appeared to foster a sense of autonomy and engagement in participants’ therapeutic process. This may be understood by the self-determination theory which emphasizes the effect of autonomy on promoting self-efficacy and intrinsic motivation [34, 35]. However, participants also expressed the importance of human connection in therapy, which was lacking in the computerised task of CBM-pa. Most of them favoured the idea of nesting CBM-pa within some form of supportive human interaction, such as the opportunity for debriefing or more formal combination with a structured talking therapy. This is in line with previous literature in which human assistance or involvement is consistently welcomed and valued by users in computerised treatments [36, 37].

### Future research and developments based on feedback

To address participants’ concerns about the test-like nature of CBM-pa and the desire for being informed in advance, a briefing that portrays CBM-pa as a ‘life simulation’ game, or similar, may be a potential solution. Participants could be instructed to role play the protagonist in the social situations presented and make interpretations of the ambiguous scenarios to find out what really happened. Explicit emphasis could be placed on the absence of absolute right or wrong answers, instead focussing participants on a goal to find out their character’s actual experience as it unfolded in the game. Featuring CBM-pa as a game in this way may reduce the test-like impression of the task without compromising the principle of self-determination theory that enables engagement and intrinsic motivation. The briefing may also, as Beard [6] suggested, normalise possible experiences of participants, such as perceptions of repetitiveness or boredom.

In terms of programme delivery, participants’ concentration may be enhanced through scheduling breaks within the session and further developing scenario texts to include matching visual or/ and audio input. Also, short alternative exercises could be incorporated in between the CBM task to sustain participants’ interest. To cater to the need of people with lower literacy level, more time could be allocated for each item in the task. Were CBM-pa to be used as an intervention in future, it would be entirely self-administered and therefore issues associated with the researcher’s presence would not arise.

CBM-pa could be combined with elements of human interaction by conducting mid and end-of-program debriefing sessions. The aim would be three-fold; to offer low-intensity emotional support; to address barriers to engagement with the intervention (such as initial resistance to interpretations that run counter to patients’ usual patterns of thought) and to address negative affect that might be elicited during sessions. Booster sessions could be considered as a maintenance strategy to augment any treatment effects. Prior studies have suggested that interventions which include booster sessions are more effective and sustainable than those without [38]. Future work is needed to examine the feasibility and efficacy of the above recommendations.

### Study limitations

There were some notable limitations in our study. First, the presence of two interviewers could be perceived as a power imbalance and increase the likelihood of socially desirable responding. However, this should be set against the advantage that the second interviewer could facilitate the interview with additional prompts. The quality of an interview often depends on how questions are phrased. This is especially true when covering distressing emotional events in interviews with mental health patients. Having an additional interviewer also helped with detecting risk of bias and enhancing the rigour of the data*.* Second, the likelihood of socially desirable responding was also increased by there being some overlap in researchers conducting interviews and supporting the delivery of the intervention. Third, despite our sample presenting with active paranoid symptoms, participants were selected to be generally stable in mental state. Thus, our findings will not be representative of those with severe persecutory delusions or in acute psychotic states. Fourth, since the researchers in the current study were also developers of CBM-pa, researcher bias may exist. Lastly, this study was only able to reflect participants’ acceptability of CBM-pa conducted under a research setting but not without the presence of a researcher. Future research is needed to ascertain its acceptability if CBM-pa were to be developed as a home-based intervention.

## Conclusions

Our study provides important user-feedback that CBM-pa is acceptable and is well-liked among patients with psychosis. Participants reported raised insight into biased paranoid interpretations and increasing capacity for considering alternative explanations of emotionally ambiguous situations. Our analysis highlighted the test-like impression created by CBM-pa in its current format, but that participants were also able to understand the task as an intervention, which proved helpful. Participants’ expressed a preference for human interaction to supplement their experience of CBM-pa. The current findings identify a number of ways to improve the design and implementation of the CBM-pa protocol. Patient opinion supports the further development and testing of CBM-pa as a treatment for paranoia.

## Additional files

Additional file 1: CBM-pa qualitative study topic guide. (DOCX 22 kb)

Additional file 2: Practical recommendations on the design of CBM-pa.doc with participants’ quotes. (DOCX 24 kb)

## Data Availability

Although anonymized, the qualitative data that supports the findings of this study are potentially identifiable and not therefore suitable for depositing in a public database. The data are however available from the authors upon reasonable request subject to ethical permissions and participant consent.

## Abbreviations

ARMS: At risk mental state
CBM: Cognitive bias modification
CBM-A: Cognitive bias modification-Anxiety
CBM-I: Cognitive bias modification-Interpretation
CBM-pa: Cognitive bias modification for paranoia
LEAP: Lived Experience Advisory Panel
PANSS: Positive and Negative Syndrome Scale
RCT: Randomized Controlled Trial
SRT: Similarity Rating Task
