# Supplementary material for: A qualitative study of the acceptability of cognitive bias modification for paranoia (CBM-pa) in patients with psychosis
Source: BMC Psychiatry. 2019 Jul 23;19:225. doi: 10.1186/s12888-019-2215-3 (PMC6651961; doi:10.1186/s12888-019-2215-3)
Supplement: Supplementary file 1 — CBM-pa qualitative study topic guide. (DOCX 22 kb) [file 12888_2019_2215_MOESM1_ESM.docx]

**Additional file 1**

**CBM-pa Qualitative interview topic guide**

1. Why did you decide to participate in the study?

- What did you hope to achieve from participating in the study?

2. What were your expectations of participating in the study?

- What did you think would be good about the study?
- What did you think would be bad about the study?

3. How did you find the computer programme in the study?

- What was helpful about the programme?
- What was less helpful about the programme?
- How do you think the programme could be improved?
- How did you find the therapy being delivered on computer?

4. How did you find the visual display of the information presented on the computer screen?

- How clear or unclear was the information?
- What did you like about the presentation of the information on the screen?
- What did you dislike about the presentation of the information on the screen?
- How would you improve the way information is presented on the computer screen?

5. What did you think of the scenarios in the computer programme?

- To what extent were you able to identify/relate to the scenarios? (easy/difficult to identify with/relate to)
- What did you think of the alternative answers offered by the computer programme?
- How would you improve the scenarios?

6. What did you think about the number and length of the sessions?

- How did you find the length of each individual session?
- What did you think about having six sessions in total?
- How would you improve the number and length of sessions?

7. How did you find the experience of completing the computer programme on your own?

- What were the advantages of this?
- What were the disadvantages of this?

8. How did this computer programme compare to other treatments or therapies that you’ve received in the past?

- What were the advantages of this programme compared to others?
- What were the disadvantages of this programme compared to others?

9. Can you give any other suggestions you have for improving the programme?

- Is there anything else you would like to tell me about the computer programme and your experience of it?
- To what extent has completing the computer programme impacted on your life? (positive/negative/no noticeable impact)
