# Supplementary material for: A qualitative study of the acceptability of cognitive bias modification for paranoia (CBM-pa) in patients with psychosis
Source: BMC Psychiatry. 2019 Jul 23;19:225. doi: 10.1186/s12888-019-2215-3 (PMC6651961; doi:10.1186/s12888-019-2215-3)
Supplement: Supplementary file 2 — Practical recommendations on the design of CBM-pa.doc with participants’ quotes. (DOCX 24 kb) [file 12888_2019_2215_MOESM2_ESM.docx]

**Additional file 2**

Practical recommendations on the design of CBM-pa

| 1. **Appeal of CBM-pa**  - Add audio and visual (video/ picture) display that matches with the scenario to enhance participants’ attention and sense of personal relevance while working on the task.   *“Yeah words is good, but also illustrations you know pictures you know, would be good as well you can get someone thinking like you know- the cloud and stuff like that, those could help as well ‘cause or like you know- illustrations that portray something that they can look at and can think- ahh yes- they can relate to, things like that you know”(Participant1)*   1. **Programme structure**  - Implement CBM-pa for a longer period (twice a week and up to six months) to consolidate learning and sustain its impact.   *“I think it’s good for building up ideas and ways of doing things and acceptable ways of doing things, but I don’t think you can achieve it in 5 weeks or 6 weeks…at least 10 [sessions] I think, yeah…every 2 weeks that takes it to half a year doesn’t it?” (Participant 6)*   - Incorporate alternative exercise in between the CBM-pa task to sustain interest   *“I think it got a bit repetitive umm the scenarios were all different but the actual methodology was the same umm so I was always glad to do something different as well, cause we…we had like a spelling test and things like that…so I like that as well…I think to break up that main block would be… good to break it up have something in the middle and then go back to it.” (Participant 6)*   - Allow extra time for response.   *“I just thinking erm like…giving people extra time to erm, to answer the questions rather than it just turning off after a couple of seconds..” (Participant 1)*   - Give participants the option of completing CBM-pa at home.   *“Maybe you can give people the option to do sometimes you can do at home or something…case they don’t have to travel here…it was okay…but maybe for some people it would help to maybe some tasks here, some at home” (Participant 5)*   1. **Content of scenarios**  - Tailor the content of scenarios to increase personal relevance to participants. For instance, add a job interview scenario for those who would like to seek for job.   *“Then it’s also I think people who want to go back to work, talking about the job interview situation, or facing criticism that sort of things I think would be helpful.” (Participant 5)*   1. **Being informed**  - Give an introduction of the intervention in advance and provide explanation of the rationale.   *“More of an introduction to it so you have a practice…but bit more of an introduction before the practice, saying what you need to do, what its developing, or what you aim to gain from it some sort of it, gain from the service users’ perspective umm, so rather than just lay into it, so introduce it and say we hope that this will improve blah blah or whatever you think appropriate” (Participant 6)*   1. **Human contact**  - Offer means of contact in case emotional support is needed   *“Yeah if there was like a bit more support like if there was someone like at the end of the phone, just to pick up in case something is getting really bad, like for instance the time I couldn’t handle my, if it had gotten any worse I would have liked to have someone to maybe pick up the phone or talk to on email or something like that” (Participant 8)* |
| --- |
